# Spatial action–effect binding depends on type of action–effect transformation

**DOI:** 10.3758/s13414-020-02013-2

**Published:** 2020-03-04

**Authors:** Marvin Liesner, Wladimir Kirsch, Roland Pfister, Wilfried Kunde

**Affiliations:** Department of Psychology, University of Würzburg, Würzburg, Germany

**Keywords:** Action–effect compatibility, Agency, Body ownership, Ideomotor theory, Proprioceptive drift, Spatial binding, Tool use

## Abstract

Spatial action–effect binding denotes the mutual attraction between the perceived position of an effector (e.g., one’s own hand) and a distal object that is controlled by this effector. Such spatial binding can be construed as an implicit measure of object ownership, thus the belonging of a controlled object to the own body. The current study investigated how different transformations of hand movements (body-internal action component) into movements of a visual object (body-external action component) affect spatial action–effect binding, and thus implicit object ownership. In brief, participants had to bring a cursor on the computer screen into a predefined target position by moving their occluded hand on a tablet and had to estimate their final hand position. In Experiment 1, we found a significantly lower drift of the proprioceptive position of the hand towards the visual object when hand movements were transformed into laterally inverted cursor movements, rather than cursor movements in the same direction. Experiment 2 showed that this reduction reflected an elimination of spatial action–effect binding in the inverted condition. The results are discussed with respect to the prerequisites for an experience of ownership over artificial, noncorporeal objects. Our results show that predictability of an object movement alone is not a sufficient condition for ownership because, depending on the type of transformation, integration of the effector and a distal object can be fully abolished even under conditions of full controllability.

## Introduction

Motor actions often aim at producing a change in the environment that does not occur in direct proximity of the effector, but rather at a more distant spatial location. This is especially the case when using a tool (e.g., when hitting a nail with a hammer or when moving a cursor with the computer mouse). Such (inter)actions give rise to a striking perceptual distortion: The perceived position of the effector is systematically shifted towards the location of the action’s effect and vice versa, and such *spatial binding* has been documented in a range of studies.

For example, when a (nonvisible) hand movement produces a visual cursor movement with an angular deviation, the perceived position of the hand shifts towards the end position of the cursor, while the perceived end position of the cursor shifts towards the end position of the hand (Debats, Ernst, & Heuer, 2017a, 2017b; Rand, Wang, Müsseler, & Heuer, 2013). Similarly, Kirsch, Pfister, and Kunde (2016) found the same mutual attraction effects when a spatial offset between uniformly, horizontally moving hand and cursor instead of an angular deviation was introduced.

The spatial binding effect has been explained as resulting from multisensory integration of sensory signals stemming from the action (especially proprioceptive and visual information) and sensory signals of the resulting effect (e.g., Kirsch et al., 2016). Indeed, similar spatial attraction effects have been observed in multisensory perception, suggesting that the currently most reliable sensory channel biases the localization of signals from other modalities (e.g., Alais & Burr, 2004). One striking example for this mechanism is the popular rubber-hand illusion (Botvinick & Cohen, 1998), in which the participant’s real hand is occluded from vision while it is softly and synchronously stroked with an artificial hand that is positioned in the participant’s view. Following synchronous stimulation, participants reliably report to “feel” the stroke no longer on their own hand, but rather on the rubber hand, and that when asked to localize their own hand, they tend to judge it closer to the rubber hand, a phenomenon now known as “proprioceptive drift” (Tsakiris & Haggard, 2005). This effect is often interpreted as an implicit proxy of experienced “ownership” (i.e., the feeling that the rubber hand is part of one’s own self or body representation; Tsakiris, 2010).

Rubber-hand illusions were also studied with an active version of the paradigm in which participants move their (occluded) real hand and observe an artificial entity to follow their movements (e.g., Dummer, Picot-Annand, Neal, & Moore, 2009; Kalckert & Ehrsson, 2014). Interestingly, several studies suggested that such procedures may induce feelings of ownership even for virtual entities that do not resemble the human body, given that the effects observed in these entities are controlled by the person and are temporally contingent to their own movements (e.g., simple geometrical objects; Ma & Hommel, 2015; Sanchez-Vives, Spanlang, Frisoli, Bergamasco, & Slater, 2010; Zopf, Polito, & Moore, 2018; but see Kalckert, Bico, & Fong, 2019). For example, for objects that move synchronously rather than asynchronously with the body, participants report larger embodiment or ownership, and show increased skin conductance in case of object threat.

The setup of these studies resembles experimental setups to study spatial action–effect binding. Here, participants are typically asked to carry out movements in a horizontal plane (e.g., on a digitizer tablet), which then cause object movements on either a vertically oriented screen or were projected onto the horizontal plane (e.g., Debats et al., 2017a, 2017b; Kirsch et al., 2016; Liepelt, Dolk, & Hommel, 2017; Rand & Heuer, 2013). Due to the high similarity of both types of experimental paradigms, it thus seems fair to assume that studies on active rubber-hand illusions and studies on spatial action–effect binding address similar processes.

Previous research suggests that a major driving force behind rubber-hand illusions and spatial action–effect binding is the detection of cross-correlations between sensory information from the manipulated object and sensory information from one’s own hand (Debats et al., 2017a). Consequently, these effects diminish or even disappear when the object is no longer contingent on the body movements (Debats et al., 2017b; Debats & Heuer, 2018b; Kalckert & Ehrsson, 2014; Kalckert, Perera, Ganesan, & Tan, 2019; Kirsch et al., 2016). Cross-correlations have typically been implemented in terms of high spatiotemporal similarity of (multimodal) sensory changes. However, for transformed movements, there are often situations in which hand movements trigger correlated events that are, however, spatially incompatible to the hand movement. This is the case, even in simple systems such as levers with one pivot point, in which hand movements in one direction (say, left) make the lever move in the opposite direction (say, right). In this situation, there is a high cross-correlation between sensory information resulting from the hand movement and sensory information relating to the lever movement, but the two movement trajectories are obviously in conflict. Studying spatial binding in this situation allows for drawing conclusions regarding the process that gives rise to perceptual distortions such as spatial action–effect binding: If the process underlying spatial binding mainly captures systematic covariation (i.e., unsigned cross-correlations), then spatial binding should also be apparent for transformed movements. If the process draws on direct spatial matching (i.e., signed cross-correlations), then spatial binding should not occur in the case of transformed movements.

We tested these hypotheses by coupling compatible or incompatible action effects to movements, which participants performed with a stylus on a digitizer tablet. That is, in the compatible condition, hand movements caused cursor movements in the same direction and of the same magnitude. In the incompatible condition, the cursor still moved to the same extent as the hand, but always in the opposite direction. Based on models of ownership it is difficult to judge whether a mismatch in the directions of hand and cursor movement results in less binding of the manipulated object and the hand (Tsakiris, 2010; Tsakiris, Carpenter, James, & Fotopoulou, 2010). These models typically focus on a lack of predictability of vision and body-related stimulation (e.g., by reducing synchronicity of hand and object movements or corresponding stimulation). In the present study, in contrast, the predictability of hand and visual object movements remains the same, and only object movement direction is manipulated. Also, models of statistically optimal integration of proprioceptive and visual information make no clear prediction of whether spatial binding would occur at all in our conditions and whether it would vary with type of transformation (when controlled for all other kinematic characteristics). Typically, studies on optimal integration use setups with only marginal, and mostly unnoticeable, discrepancies between proprioceptive and visual information (e.g., Debats et al., 2017a, 2017b; Kirsch et al., 2016). In contrast, in the current study, proprioceptive and visual information were clearly separated, rendering it uncertain whether integration of multimodal signals would occur at all. Previous studies showed some heterogeneous findings regarding the integration of more or less far apart sensory signals. Debats and Heuer (2018a), for example, found that knowledge of spatial disparity of the sources of visual and proprioceptive feedback can reduce sensory integration, while Misceo, Jackson, and Perdue (2014) suggested that explicit knowledge about a common cause of the sensory signals did not increase integration. Furthermore, Rand and Rentsch (2016) showed that even with spatial disparities of up to 150° between an external cursor and the hand, integration of sensory signals can still occur in a way that the estimation of the actual hand location was biased towards the cursor. However, the aim of this latter study was actually to investigate adaptation effects to feedback rotations, so it remains unclear how or if this finding would generalize to other settings, and especially to the case of fully inverted action–effect relations.

As a second aim, we intended to further examine the similarity of rubber-hand illusions and spatial binding by introducing explicit ratings of sense of ownership (i.e., the feeling that the manipulated object belongs to one’s own body) to the study of spatial action–effect binding. That is, while studies on the rubber-hand illusions often included explicit measures of agency (i.e., the feeling of control over the object movement) and ownership alike (e.g., Kalckert & Ehrsson, 2014), studies with more simple action effects have mainly focused on the former ratings. Such explicit ratings of agency were reported to be lower for spatially incompatible action–effect transformations than for spatially compatible ones (Ebert & Wegner, 2010; Liesner, Kirsch, & Kunde, 2020). Our goal was to replicate these findings for agency and to assess whether ratings of ownership, though perhaps relatively low for such artificial objects in the first place, would be affected by action–effect transformations in the same way as spatial binding. Finally, action–effect (in)compatibility is often found to affect performance as well (e.g., in terms of reaction times; e.g., Kunde, 2001; Kunde, Pfister, & Janczyk, 2012; Pfister & Kunde, 2013). Therefore, we also expected to find faster reactions when hand movements would produce spatially compatible rather than incompatible action effects.

## Experiment 1

**Fig. 1:**
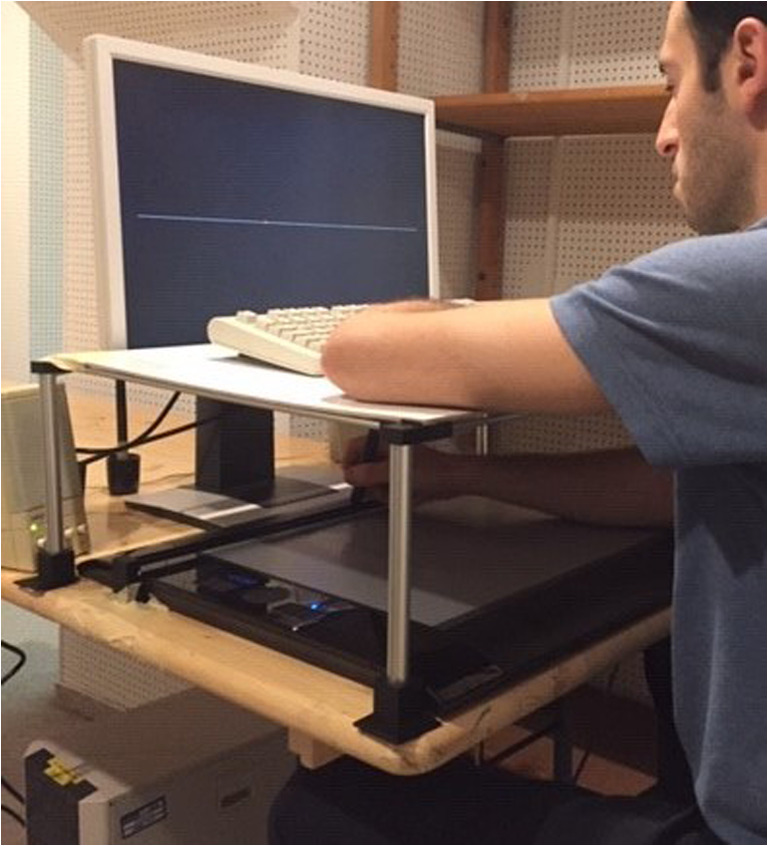
The experimental setup used in both Experiment 1 and Experiment 2

Experiment 1 targeted the influence of different action–effect transformations on spatial action–effect binding, reaction times, and explicit ratings of agency and ownership by employing a transformed mouse-cursor movement. In the most relevant conditions, participants were asked to move a cursor on a screen to a certain target position while hand and cursor movements were either spatially compatible or spatially incompatible to each other (cf. Fig. 1). When cursor and hand were in the target position, participants were asked to judge the position of the cursor in one condition, and the position of the hand in another condition. Importantly, the positions of hand and cursor at the point of judgments were identical in both compatibility conditions. The only difference was whether these positions had been reached by a spatially compatible or spatially incompatible transformation of hand-to-cursor movement. We also added a baseline condition in which the cursor was always presented at the same position as the hand. This allowed us to control for possible general tendencies or biases in the spatial judgments. In a subset of trials, we further collected explicit measures of agency and ownership to compare them with the implicit measure of spatial action–effect binding.

In addition to these judgments, we asked participants to begin each trial with an initial cursor movement to a left or right target box, depending on cursor color. This was meant to familiarize participants with the (in)compatibility of the movement transformation in the given trial and to reveal potential conflict between hand movement and cursor movement during action production, which should be reflected in overall increased response times with incompatible rather than compatible hand–cursor transformation (e.g., Kunde et al., 2012; Schwarz, Pfister, Wirth, & Kunde, 2018).

## Material and methods

### Ethics statement

The present experimental paradigm was approved by the ethics committee of the Institute for Psychology of the University of Würzburg under the reference number GZEK 2018-33. All procedures were in line with the Declaration of Helsinki.

### Participants

Thirty-nine participants were tested in Experiment 1 and received either course credit or €10 per hour. Participants were recruited through an online platform used by the University of Würzburg and gave informed consent prior to the experiment. Visual inspection of the data suggested that three participants had obviously misunderstood the task, because their spatial judgments were consistently much closer to the not-to-be-judged position (hand vs. cursor) than the position they were meant to judge. These participants were excluded from analyses so that the final sample contained 36 participants (27 female, nine male; all right-handed; *M*_age_ = 27.81 years, *SD*_age_ = 9.26 years, *min*_age_ = 19 years, *max*_age_ = 65 years).

The sample consisted of two subgroups, one of which had to judge the position of the cursor upon reaching the final position, whereas the other group had to judge the position of the hand, or, more precisely, the stylus in their hand. Assuming an effect size of *d*_*z*_ = .77 as observed for the smaller effect on cursor judgment in Kirsch et al. (2016), a power analysis suggested a sample size of 16 participants for a power of .8. To keep order of conditions and stimulus–response mappings counterbalanced between all participants, we thus decided to test 18 participants per judgment condition.

### Apparatus and stimuli

Figure 1 shows a photograph of the experimental apparatus. The setup consisted of a graphics tablet (Intuous 4 XL, Wacom, Kazo, Saitama, Japan), which was horizontally fixated on a table and placed under an additional board to prevent vision of the tablet, a digitizing stylus to operate the tablet, a 22-in. LCD monitor to present stimuli and ratings, and a keyboard placed on top of the occluding board. The experimental procedures were programmed using E-Prime (Version 2.0, https://www.pstnet.com/). The tablet and the occluding board were placed at the edge of the table, while the monitor was placed in a central position behind the tablet and board (around 46.2 cm from the front edge of the table). Furthermore, two additional plastic bars were attached to the upper part of the tablet, forming a rail for the digitizing stylus to prevent participants from moving the stylus in the direction of the *y*-axis of the tablet. This rail was placed as close to the top of the tablet as possible to minimize the actual physical distance between the stylus on the tablet and the stimuli presented on the monitor. The height of the monitor was adjusted so that the bottom of the screen was approximately at the same level as the occluding board to ensure that visual features of the monitor (e.g., buttons or letters printed on its frame) could not serve as supporting landmarks for the spatial judgments, and the boundaries of the active surface of the tablet were set so that they resembled the left and right boundaries of the screen. One pixel of the monitor was approximately 0.47 × 0.47 mm^2^ in size. Finally, the keyboard was placed on top of the occluding board. Participants were seated in front of the setup with their left arm laying on the occluding board, allowing them to use the keyboard, and their right arm placed on the tablet with the digitizing stylus in their hand. They could freely adjust the height of the chair and its proximity to the table to make sure that participants were sitting in a position from which they could properly perform all parts of the experiment. All participants used the right hand to move the stylus on the digitizing tablet.

### Procedure and task

Each trial included the following steps for the participants. First, participants had to move the cursor (a little green dot, approximately 1.40 mm in diameter) on a grey horizontal line on the screen until the center of the screen was reached, as marked by another yellow dot, equal in size. Reaching the screen center made two target boxes appear approximately 86 mm to the left and right of the center position. After a predefined delay of either 1,000 ms or 2,000 ms (in 50% of all trials each and counterbalanced over all conditions), an auditory signal (50 ms, 2000 Hz) was played, and the cursor changed its color to either blue or orange, which indicated whether the cursor had to be moved into the left or right target box (color–response mapping was counterbalanced between participants). Participants were instructed to respond to the color change as fast as possible. When the correct target box was reached with the cursor, it turned grey to provide feedback to the participant of successful completion of this task. When participants moved the cursor to the wrong side or showed no reaction within 1,500 ms after stimulus onset, an error message was shown, and the trial was repeated. In all conditions and also in all conditions including reaction-time tasks in Experiment 2, reaction times were measured as the time interval from stimulus onset until the stylus had been moved 1.7 mm either to the left or the right. After another delay of 1,000 ms, both target boxes disappeared, and the participants’ task was to move the cursor on the horizontal line until it turned red at one out of six predefined positions, and then maintain this position. These positions were spread out on the entire horizontal line and could be more lateral than the target boxes that had to be reached for in the first reaction-time task. Thus, the final movement of the hand towards the estimation position could be both a movement towards the center of the tablet or further towards its sides. By doing so, there was an equal number of hand movements towards the cursor or away from it in both the additive and inverted mapping. After pressing a button on the stylus, both the cursor and the horizontal line disappeared, and after a delay of 1,000 ms, a red vertical line (about 17 mm long) appeared at the top of the screen about 110 mm above the previous height of the horizontal line and with a displacement from the to-be-judged position (cursor vs. hand) of 17 mm to the left or right varying randomly. The final task of the participants was to adjust the vertical line by pressing the left and right arrow keys with their left hand so that the line would “point” down on either the position where the cursor had just disappeared or the position where they perceived the tip of the stylus in their hand to be. Whether the stylus position or the cursor position had to be judged was manipulated between participants so that half of the participants always judged the cursor position and the other half always judged the stylus position. Importantly, during the whole process of the spatial judgment, participants had to maintain the stylus and cursor at the same position or an error message was shown and the trial was repeated. Additionally, every 12 trials, participants had to state on a 9-point Likert scale (without anchor or label values; Likert, 1932) how much they had the impression to control the cursor (agency rating) and how much they had the impression that the cursor was part of their body (ownership rating).

**Fig. 2:**
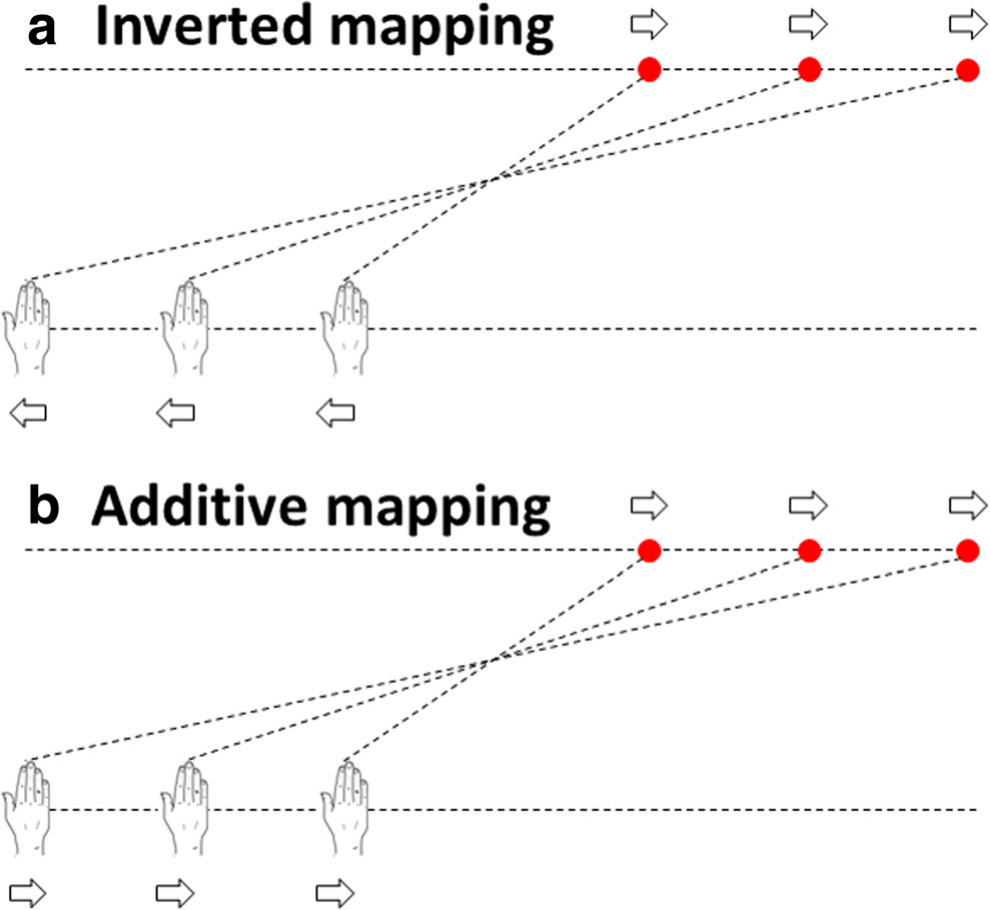
Action–effect mappings and final positions of hand and cursor during judgments in the two main conditions of both experiments. Hand movements were either transformed to yield inverted cursor movements (**a**) or they were displayed with an additive horizontal offset (**b**). Arrows indicate the direction of the movement of hand and cursor. In the inverted condition, the cursor always moved in the opposite direction than the hand while it moved in the same direction in the additive condition. The offsets between hand and cursor in the additive condition were chosen in a way that the end point of hand and cursor always resembled those in the inverted condition. Thus, there were three different offset sizes either to the left or to the right in the additive mapping so that the end positions of both hand and cursor would always exactly reflect those in the inverted mapping, where the cursor behaved mirror-symmetrically to the hand. Experiment 1 further included a baseline condition with direct mapping of hand and cursor position, whereas Experiment 2 introduced two additional control conditions in which the participants did not control the cursor

The experiment consisted of three types of blocks, which alternated the mapping with which the stylus movements were translated into cursor movements on the screen. The order of blocks was counterbalanced across participants. In blocks with *direct mapping,* the horizontal cursor position always exactly reflected the position of the stylus (i.e., their *x* coordinates were equal). In blocks with *inverted mapping,* the horizontal cursor always reflected a centrally mirrored position of the stylus in the plane. Thus, a movement of the stylus to the left or right from the center of the tablet always resulted in a movement of the cursor to the same extent in the opposite direction from the central position (see Fig. 2). Finally, in blocks with *additive mapping,* the cursor always moved in the same direction as the stylus, like in the direct mapping, but there was always a certain, constant offset either to the left or to the right between the cursor and the stylus in every trial. There were six different kinds of offset, which were chosen in such a way that the final distance between stylus and cursor when reaching the position for the spatial judgment was always identical to the difference in the inverted mapping when the cursor had to be brought to the same position (see Fig. 2).

Each block consisted of 48 trials, with every combination of all manipulated factors (judgment position: six levels; required response left/right in reaction-time task: two levels; ISI: two levels; offset of vertical line for judgment left/right: two levels) presented once. Order of blocks was counterbalanced across participants, and before every block there were 12 practice trials so that participants could get used to the stylus–cursor mappings. Overall, participants thus completed 180 trials plus possible repetition trials due to mistakes. Per participant, the experimental session took approximately 60–75 minutes.

### Data preprocessing

We excluded all practice trials and all trials with any kind of errors that were replaced by repetitions of that exact same trial. For every judgment of cursor or stylus position, we computed the estimation error by calculating the difference between the estimated position and the actual position. Thus, negative values for estimation errors represent a judgment that is more to the left than the actual position, and positive values for estimation errors represent a judgment that is more to the right than the actual position.

## Results

All raw data and analysis scripts are available on the Open Science Framework (osf.io/tgd95/).

### Spatial binding

Median estimation errors were analyzed separately for cursor and stylus judgments. We calculated separate 3 × 6 repeated-measures (RM) analyses of variance (ANOVAs), with the factors mapping (additive, inverted, direct) and final stylus or cursor position during judgment (six levels) for both kinds of judgments, respectively. In cases of violation of the sphericity assumption, Greenhouse–Geisser-corrected *p* values and the correction factor ε are reported (Greenhouse & Geisser, 1959), accompanied by uncorrected degrees of freedom.

**Fig. 3:**
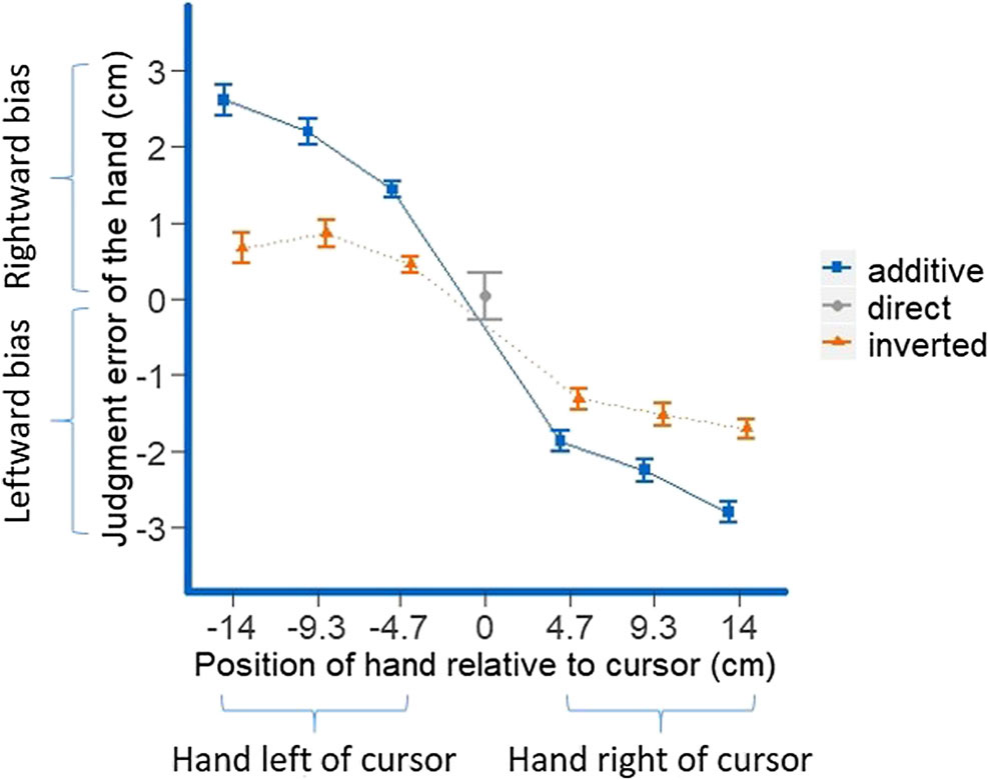
Mean estimation errors for all three mappings and all final stylus and hand positions. Note that in the direct condition, the cursor was always right above the hand, and so the difference between the hand and cursor coordinate was always zero. Negative values for the relative cursor position indicate that the final cursor position is to the right of the hand and positive values for the relative cursor position indicate that the final cursor position is to the left of the hand. Positive values for the judgment error indicate judgment errors of the hand to the right and negative values for the judgment error indicate judgment errors of the hand to the left. Error bars for the additive and inverted conditions represent 95% confidence intervals of paired differences between the two conditions (Pfister & Janczyk, 2013) and for the direct condition they represent the standard error of the mean. Please note that the actual relative cursor/hand positions were equidistant with an increase of 4.658 cm between cursor and hand for each position further away from the center of the tablet. Due to rounding, this equidistance is not reflected exactly in the figure

Figure 3 shows means of median estimation errors for stylus judgments. There was no significant main effect of mapping (*p* = .31); however, both the main effect of stylus position, *F*(5, 85) = 18.48, *p* < .001, ε = .25, η_p_^2^ = .52, and the interaction between the two factors, *F*(10, 170) = 23.74, *p* < .001, ε = .37, η_p_^2^ = .58, were significant. To unravel this interaction, we first assessed possible general biases towards the left or the right or for certain stylus positions by testing the median estimation errors for the direct mapping against zero, which revealed no judgment errors different from zero for any hand position (all *p*s > .67, two-tailed). To analyze the differences between the additive and inverted mapping, we computed separate linear regressions for every participant for each of the two mappings, with the difference in horizontal direction between stylus and cursor during judgment as predictor and estimation error as criterion. We then compared the slopes for the regressions for both mappings (Lorch & Myers, 1990), which revealed a significantly more negative slope for the additive than for the inverted condition (*M*_β additive_ = −.68, *SD* = .29; *M*_β inverted_ = −.45, *SD* = .42), *t*(17) = 3.29, *p* = .002, one-tailed, *d*_*z*_ = .77. This suggests that the attraction of the judged stylus position towards the cursor is stronger in the additive than in the inverted condition, both when the hand with the stylus is to the left of the cursor (negative difference between stylus and cursor, positive values for estimation error) and when it is to the right of the cursor (positive difference between stylus and cursor, negative values for estimation error). Please note that while the second, six-level factor in the analysis was the actual stylus position on the tablet during judgments, in Fig. 3 the distance between the stylus (or the hand holding it) and the cursor are plotted on the *x*-axis to represent the relationship between hand–cursor distance and estimation error. Therefore, the direct condition is only represented by a single dot in Fig. 3, as the distance between cursor and stylus/hand was always zero in this condition.

The same 3 × 6 RM ANOVA for cursor judgments (now the second six-level factor representing the final cursor position) did not reveal any significant effects (all *p*s > .065).

Additionally, we also performed exploratory analyses for nonlinear (i.e., quadratic and cubic) regression components. However, neither the quadratic nor the cubic regression coefficients significantly improved the fit for any of the two relevant conditions, though there were reasonable descriptive cubic influences in both conditions (both βs > .30, both *p*s < .18) However, these coefficients also did not differ between conditions.

### Agency/ownership ratings

Mean agency and ownership ratings were entered into a 3 × 2 split-plot ANOVA, with the within-subjects factor mapping (additive, direct, inverted) and the between-subjects factor judgment type (cursor vs. stylus). We found a significant main effect of mapping for agency ratings, *F*(2, 68) = 4.66, *p* = .016, ε = .89, η_p_^2^ = .12. Experienced agency was higher for the direct (*M* = 6.79, *SD* = 1.93) than for the inverted (*M* = 6.08, *SD* = 1.89) *t*(35) = 3.09, *p* = .002, one-tailed, *d*_*z*_ = .51, and the additive mapping (*M* = 6.40, *SD* = 1.78). *t*(35) = 1.99, *p* = .027, one-tailed, *d*_*z*_ = .33, while there was no difference between the latter two (*p* = .11, one-tailed). The main effect of mapping for experienced ownership did not reach significance (*p* = .26), and neither did all other effects for both kinds of ratings (all *F*s < 1; see Table 1).

**Table 1:** Means (and standard deviations) of ratings in Experiment 1 for all mappings

		Mapping		
		Direct	Additive	Inverted
Rating type	Sense of agency	6.79 (1.93)	6.40 (1.78)	6.08 (1.89)
	Sense of ownership	3.91 (1.95)	3.71 (2.18)	3.55 (2.10)

### Reaction times

Mean reaction times (RTs) were entered into a 3 × 2 × 2 split-plot ANOVA, with the within-subjects factors mapping (additive, direct, inverted) and required response (left or right), and the between-subjects factor judgment type (cursor or stylus).

We found a significant main effect of mapping, *F*(2, 68) = 15.16, *p* < .001, ε = .86, η_p_^2^ = .31, which was modulated by a significant interaction between mapping and judgment type, *F*(2, 68) = 5.10, *p* = .012, η_p_^2^ = .13, so we analyzed the RTs of the different mappings separately for the two judgment conditions. For the cursor judgment condition, RTs were slower for the inverted (*M* = 739 ms, *SD* = 127 ms) than for the direct (*M* = 657 ms, *SD* = 120 ms), *t*(17) = 5.41, *p* < .001, one-tailed, *d*_*z*_ = 1.28, and additive mapping (*M* = 658 ms, *SD* = 110 ms, *p* < .001, one-tailed, *d*_*z*_ = 1.02), while there was no difference between the latter two, *t*(17) =.050. For the stylus judgment condition, there were no significant differences in RTs for the three mappings (all *p*s > .068, one-tailed; see Table 2). None of the remaining effects was significant (all *p*s > .25).

**Table 2:** Means (and standard deviations) of reaction times in Experiment 1 for all combinations of mapping and judgment type

		Mapping		
		Direct	Additive	Inverted
Judgment condition	Cursor	657 ms (120 ms)	658 ms (110 ms)	739 ms (127 ms)
	Stylus	701 ms (147 ms)	690 ms (164 ms)	717 ms (163 ms)

## Discussion

In Experiment 1, we investigated how different kinds of action–effect transformations affect reaction times in a forced-choice task, explicit ratings of agency and ownership, and, most importantly, spatial binding between the participant’s hand used to control an object and the object itself. We observed that proprioceptive drift of the felt hand position towards the cursor was larger in the additive condition where hand movements and object movements were spatially compatible than in the inverted condition where they were incompatible. Thus, there is a stronger integration of body and controlled object in the case where body and controlled object move into the same direction as when they move into different directions. Importantly, these observations can be explained neither by different distances between hand and cursor nor by different levels of covariation or contingency between hand and cursor movement, which were both identical in the additive and inverted condition. Moreover, they cannot be explained by differences in the final movement direction towards or away from the cursor just before the spatial judgments had to be made.

Interestingly, the pattern of results for the explicit agency and ownership ratings did not mirror the results of the spatial judgments, since agency ratings did not differ between the additive and inverted conditions, and both of these ratings were lower than the ones for the direct condition. Additionally, there were no differences at all between the ownership ratings for different conditions. These results add to previous studies which showed diverging patterns of results for explicit and implicit measures of agency (such as temporal binding; Buehner, 2012; Kirsch, Kunde, & Herbort, 2019; Majchrowicz & Wierzchoń, 2018; Moore, Lagnado, Deal, & Haggard, 2009; Schwarz, Weller, Klaffehn, & Pfister, 2019; Suzuki, Lush, Seth, & Roseboom, 2019) and extend these findings to the spatial domain and measures of ownership as well.

It should be noted that despite the strong binding effect that we found for stylus judgments, we found no drift of the cursor judgments towards the hand. Previous studies suggested that the visual drift is often substantially smaller than the proprioceptive drift and that it might also be more restricted regarding the conditions under which it occurs (Debats et al., 2017b; Debats & Heuer, 2018b, 2018c; Kirsch et al., 2016; Rand & Heuer, 2013). One possibility for why we did not find significant effects of visual drift towards the cursor in Experiment 1 could be that the final cursor position was very salient because participants carefully had to search for this position and the judgment task was thus rather easy. Another possible reason is that the visual drift was too weak to be detected under the present conditions.

Finally, we found the expected impeding influence of the inverted action–effect transformation on reaction times in the condition where the cursor position had to be judged, but only a trend in the expected direction in the stylus judgment condition. Conceivably, the blocked manipulation of the judgment type led participants to devote less attention to the cursor in stylus judgment blocks. Moreover, they might have recoded the imperative stimuli in the response-time task in terms of required hand movements rather than of required cursor movements. The less attention is devoted to cursor movements, the less likely is inversion of hand and cursor movement to affect action generation (see Ansorge, 2002, for similar observations). Note, though, that we obtained a compatibility influence on RTs in stylus judgment conditions of Experiment 2, allowing for the possibility that the lack of the compatibility effect might be a Type II error (see below).

## Experiment 2

Experiment 1 revealed reduced spatial binding for spatially incompatible as compared with spatially compatible action–effect transformations. This result, however, leaves open whether and to what extent an integration of cursor and hand occurred in the incompatible condition. Kirsch et al. (2016), for instance, observed that in the absence of control over an object there can still be a tendency towards this object when estimating the hand position. Such an effect can have several origins, which, however, are not related to perceptual action–effect binding. For example, it could be that because of the generally larger uncertainty related to proprioceptive than to visual feedback, the cursor might have served as an anchor for the judgment of the stylus position. Consider, for example, a situation in which you walk into your dark living room at night, and you are to locate pieces of furniture with the only available visual information being a light from your TV, in one corner of the room. Even though unrelated to the furniture you wish to locate, it is likely that you will use this light as an orientation point for your estimation, which might also lead you to estimate the piece of furniture as being closer to that light than it actually is. Thus, to assess the extent of integration in the incompatible condition, it would be important to contrast it with a condition without any control over the object to be able to distinguish between such fully perceptional biases in spatial judgments and biases due to the action–effect transformation. This was done in Experiment 2.

There were two main differences between Experiment 1 and Experiment 2. First, we decided to drop the condition where participants had to judge the cursor. So, all participants of Experiment 2 had to judge the final stylus position. Second, and more importantly, we added two additional baseline conditions in which participants did not control a visual object, because it was stationary and thus not affected by stylus movements at all.

## Materials and methods

For brevity, we will only focus on the differences to Experiment 1 here.

### Participants

Eighteen new participants (11 female, seven male; all right-handed; *M*_age_ = 26.72, *SD*_age_ = 6.61, *min*_age_ = 20, *max*_age_ = 51) were tested in Experiment 2 and received either course credit or €10 per hour for their participation. All participants were recruited through the same online platform as in Experiment 1 and again had to give their informed consent before participating.

### Apparatus and stimuli

All hardware, software, and additional equipment were the same as in Experiment 1.

### Procedure and task

As stated above, no cursor judgments had to be made in this experiment, but all participants judged the stylus position. Experiment 2 was split into two parts, the order of which was counterbalanced between participants. The first part was essentially the same as in Experiment 1, except that all three blocks were shortened to 24 trials. Furthermore, the practice blocks before each of the blocks were also shortened to six trials.

The second part of Experiment 2 introduced two baseline conditions to the paradigm. Therefore, the reaction-time task was dropped in this block, and only the grey horizontal line and a red dot were presented at one of the six judgment positions in every trial. Importantly, however, this dot was stationary and under no control of the participant. The participants’ task was then to slowly move the stylus on the digitizer tablet (without visual feedback on the screen) until a short beep (50 ms, 2000 Hz) indicated that the final position was reached, which had to be maintained. Subsequently, participants judged the position of the stylus as in Experiment 1. There were two blocks within this part of the experiment, which we will shortly describe in the following. In the *direct baseline condition,* the stylus always had to be moved so that its position on the *x*-axis of the tablet would correspond to the horizontal position of the dot on the screen, like in the direct mapping condition. In the *additive-inverted (AI) baseline condition*, the stylus had to be moved to the same position where the stylus would be needed to be moved in the original additive and inverted mapping conditions. In both blocks, the participants were not informed about the target position of the stylus before they had reached it. Thus, despite the elimination of control over the dot in the baseline conditions and it essentially being unrelated to the participants’ task, the spatial relations between cursor and stylus were identical to the inverted and additive conditions. Each baseline block consisted of 24 trials, while there were no practice trials for the baseline blocks. Overall, participants thus completed 138 trials, including practice trials, which took approximately 60–75 minutes.

## Results

### Spatial judgments

**Fig. 4:**
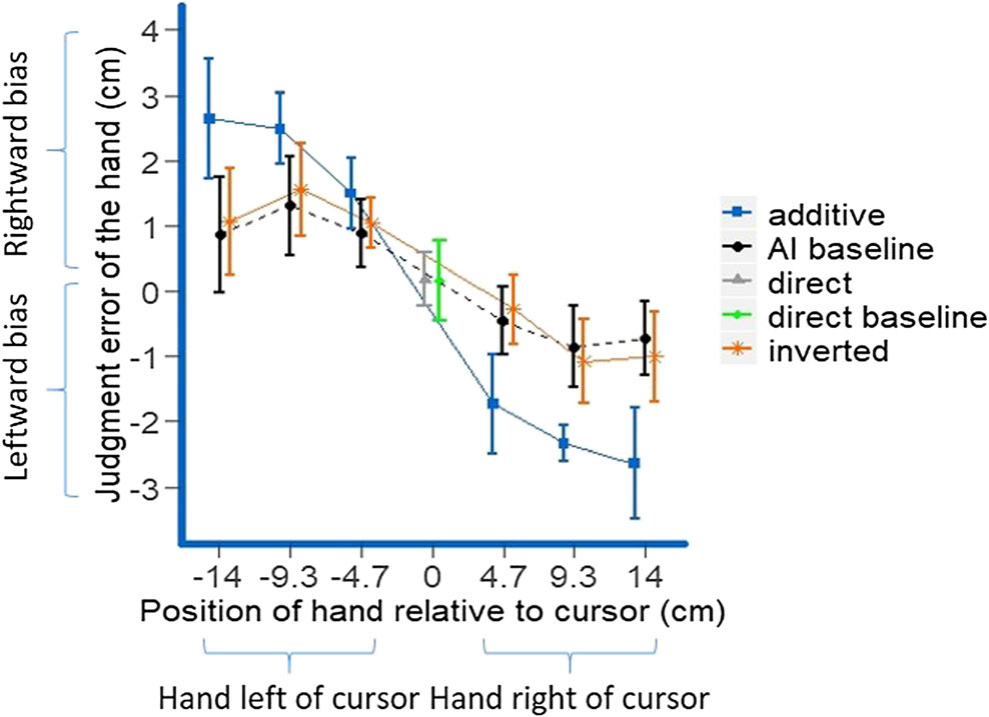
Mean estimation errors for all five mappings and all final stylus and hand positions. In the direct condition and the direct baseline condition, the cursor was always right above the hand, and so the difference between the hand and cursor coordinate was always zero. Negative values for the relative cursor position indicate that the final cursor position is to the right of the hand and positive values for the relative cursor position indicate that the final cursor position is to the left of the hand. Positive values for the judgment error indicate judgment errors of the hand to the right, and negative values for the judgment error indicate judgment errors of the hand to the left. Error bars for the additive, inverted, and AI baseline conditions represent within-subjects confidence intervals calculated separately for each final stylus and hand position (Loftus & Masson, 1994), error bars for the direct condition and the direct baseline conditions represent standard errors of the mean. Again, actual relative cursor/hand positions were equidistant with an increase of 4.658 cm between cursor and hand for each position further away from the center of the tablet

Median estimation errors were analyzed using a 5 × 6 RM ANOVA, with the factors mapping (direct vs. inverted vs. additive vs. AI baseline vs. direct baseline) and stylus position during judgment. There was no significant main effect of mapping (*F* < 1); however, there was a significant main effect of stylus position, *F*(5, 85) = 22.70, *p* < .001, ε = .28, η_p_^2^ = .57, and most importantly also a significant interaction between the two factors, *F*(20, 340) = 13.06, *p* < .001, η_p_^2^ = .43. To unravel this interaction, we first assessed possible general biases towards the left or the right or for certain stylus positions by testing the median estimation errors for the direct mapping and the direct baseline condition against zero, which revealed no judgment errors different from zero for one of the mappings at any hand position (all *p*s > .30, two-tailed). To analyze the differences between the remaining conditions, we computed separate linear regressions for every participant for the additive, inverted and AI baseline condition, with the horizontal difference between stylus and cursor during judgment as predictor and estimation error as criterion. We then compared the slopes for the regressions for the three mappings which revealed a significantly more negative slope for the additive condition (*M*_β additive_ = −.70, *SD* = .27) than for the inverted (*M*_β inverted_ = −.32, *SD* = .42), *t*(17) = 5.57, *p* < .001, one-tailed, *d*_*z*_ = 1.31, and the AI baseline condition (*M*_β AI baseline_ = −.35, *SD* = .39), *t*(17) = 5.14, *p* < .001, one-tailed, *d*_*z*_ = 1.21, while there was no difference between the latter two (|*t|* < 1; see Fig. 4). As in Fig. 3, please note that the second, six-level factor in the analysis was the stylus position on the tablet during judgments, while the *x*-axis of Fig. 4 shows the distance between the stylus (or the hand holding it) and the cursor to represent the relationship between hand–cursor distance and estimation error. Therefore, the direct condition and the direct baseline conditions are also only represented by single dots in Fig. 4; the distance between cursor and stylus/hand was always zero in these conditions.

Again, we also tested for possible nonlinear influences and found that adding a cubic component led to significantly better predictions of the criterion in the additive condition (β = .40, *ΔR*^2^ = .022, *p* = .044), but not in the other two conditions (both *p*s > .24). However, again, none of the nonlinear parameters significantly differed from each other when comparing single regression coefficients between conditions (all *p*s > .17).

### Ratings

Ratings were analyzed by pairwise *t* tests comparing agency and ownership ratings separately for the three mappings direct, inverted, and additive. Since there was no cursor in the baseline conditions, but just a stationary dot, we thus also did not assess any agency and ownership measures in the baseline condition. Experienced agency was lower for the inverted (*M* = 6.39, *SD* = 2.34) than for the direct (*M* = 7.15, *SD* = 2.17), *t*(17) = 2.65, *p* = .008, one-tailed, *d* = .62, and the additive mapping (*M* = 7.19, *SD* = 1.96), *t*(17) = 1.99, *p* = .009, one-tailed, *d* = .62, while there was no difference between the latter two (|*t|* < 1). The same pattern of results was observed for experienced ownership (*M*_direct_ = 4.42, *SD* = 2.73, *M*_inverted_ = 3.29, *SD* = 2.15, *M*_additive_ = 4.24, *SD* = 2.64), direct versus inverted: *t*(17) = 2.65, *p* = .018, one-tailed, *d*_*z*_ = .54; additive versus inverted: *t*(17) = 1.93, *p* = .035, one-tailed, *d*_*z*_ = .45; direct vs. additive: |*t|* < 1 (Table 3).

**Table 3:** Means (and standard deviations) of ratings in Experiment 2 for all mappings

		Mapping		
		Direct	Additive	Inverted
Rating type	Sense of agency	7.15 (2.17)	7.19 (1.96)	6.39 (2.34)
	Sense of ownership	4.42 (2.73)	4.24 (2.64)	3.29 (2.15)

**Table 4:** Means (and standard deviations) of reaction times in Experiment 2 for all mappings

Mapping		
Direct	Additive	Inverted
632 ms (76 ms)	638 ms (81 ms)	708 ms (93 ms)

### Reaction times

Reaction times were analyzed by means of a 3 × 2 RM ANOVA with the factors mapping (direct, inverted, additive) and required response (left or right). Since there was no reaction times task in the baseline blocks, these were also not included in the analysis. We found a significant main effect of mapping, *F*(2, 34) = 10.26, *p* = .001, ε = .82, η_p_^2^ = .38. RTs were slower for the inverted (*M* = 708 ms, *SD* = 93 ms) than for the direct (*M* = 632 ms, *SD* = 76 ms), *t*(17) = 3.88, *p* < .001, one-tailed, *d*_*z*_ = .92, and additive mapping (*M* = 638 ms, *SD* = 81 ms, *p* = .002, one-tailed, *d*_*z*_ = .77), while there was no difference between the latter two (|*t|* < 1). Neither the main effect of required response nor the interaction between the two factors reached significance (both *p*s > .055; Table 4).

## Discussion

Experiment 2 replicated the findings of Experiment 1 regarding the difference in proprioceptive drift towards the cursor between the additive and the inverted condition. Crucially, we found no difference in proprioceptive drift between the inverted condition and a baseline condition with identical physical distances between hand and cursor. This outcome suggests that there was indeed no binding for the spatially incompatible action–effect transformations and that the remaining proprioceptive drift reflects action-unrelated mechanisms, and is possibly due to the previously mentioned anchor effect. Another explanation for the differences in proprioceptive drift between conditions could be that people are used to integrate representations of their hand and a mouse cursor on a computer screen due to preexperimental experience with correlated hand and cursor movements. Construed that way, there might be a “default” coupling of hand and cursor based on long-term experience, while the additional short-term experiences from the correlations of hand and cursor movements made in the experiment are modulating this general integration tendency (for similar arguments, cf. Debats & Heuer, 2018b; Dogge, Custers, Gayet, Hoijtink, & Aarts, 2019: Wirth, Steinhauser, Janczyk, Steinhauser, & Kunde, 2018). If the long-term and short-term experiences overlap like in the additive condition, this would then lead to a stronger integration, while contradicting long-term and short-term experiences in the inverted condition should lead to a decrease in this default coupling.

Interestingly, we observed a different pattern for the explicit ratings in Experiment 2 than we observed in Experiment 1. While in Experiment 1 explicit ratings of ownership for the cursor were essentially unaffected by the different mappings and explicit agency ratings were higher in the direct condition than in both other conditions, in Experiment 2 the inverted condition yielded significantly lower ratings for both agency and ownership. This inconsistency might be due to different reliabilities of the estimations of mean values for the ratings in different conditions because the number of trials in which ratings had to be given was reduced from four to two per condition in Experiment 2 (in both experiments, ratings had to be given every 12 trials).

Furthermore, we found significant reaction-time effects in Experiment 2, which had not been significant in the stylus judgment condition in Experiment 1. Given the generally strong influence that spatially incompatible action–effect transformations usually have on action generation with the type of continuous movements we used here (e.g., Kunde et al., 2012; Müsseler, Kunde, Gausepohl, & Heuer, 2008; Müsseler & Skottke, 2011), we believe these results of Experiment 1 might have reflected a statistical Type II error.

## General discussion

The present study investigated how the integration of a noncorporeal object in terms of spatial binding is shaped by the way that the individual’s movements are transformed into movements of the object. There has been an increasing interest in this field of research in the past years, and multiple studies could show that objects, which are under full control of an individual, tend to lead to a mutual attraction of the perceived location of the object and the effective hand of the individual (Debats et al., 2017a; Debats & Heuer, 2018b; Kirsch et al., 2016; Rand & Heuer, 2013). These biases are reduced when the magnitude of control and thus cross-correlation of sensory signals decreases. For example, Debats et al. (2017a) observed less binding for curved cursor movements than for straight cursor movements when hand movements were straight. The results from the present study extend this research for several reasons. First, they demonstrate that spatial action–effect binding can be completely eliminated in situations of full and immediate control over an object when the movement direction of the object is reversed to the movement direction of the effector. Thus, controllability of an object is a necessary, but not a sufficient condition for such binding. The interesting question, however, is why an inverted movement transformation should eliminate integration of a distal controlled object and the effector controlling that object, or speaking in sensory terms, lead to less attraction of visual and proprioceptive signals.

Second, we held all other kinematic characteristics of the cursor and hand movements equal between the additive and inverted condition, except the movement direction itself: actual physical distance at judgment positions and thus “usability” of the cursor position for hand position judgments, equal gain of hand and cursor movement, controllability of cursor, and whether the hand was moving towards or away from the cursor before the spatial judgments did not differ between the two conditions. Previous studies that observed reduced mutual attraction of spatial hand and cursor judgments with increasing mismatch between hand and object movements did not control for all of these confounds (e.g., Debats et al., 2017a; Rand & Rentsch, 2016). It might be possible, though, that differences in binding resulted from different exposure times to the hand and cursor movements in the different conditions. We cannot ultimately rule out this possibility, as we did not record the time it took to complete trials. Informal observation of participants’ behavior during testing provided at least no obvious cue for differences in this respect.

Third, Debats et al. (2017a) speculated that decreases in spatial binding with discrepancies in visual and proprioceptive feedback could be related to the experience of agency. We were able to test this assumption, at least on a correlational basis, in the present study and found inconsistent results regarding the explicit ratings of agency and ownership. Thus, it has to remain unclear whether and/or to what extent the conscious experience of these conditions might have influenced the extent to which body-related and body-external signals were integrated in the present study.

Ideomotor models of human action control (e.g., Hommel, 2013; James, 1890/1981; Koch, Keller, & Prinz, 2004; Shin, Proctor, & Capaldi, 2010; Waszak, Cardoso-Leite, & Hughes, 2012) provide another view on our findings and possibly also on previous findings (e.g., Debats et al., 2017a; Rand & Rentsch, 2016) that found reduced multisensory integration, or reduced integration of a body effector and an external object. Ideomotor theory states that action–effect incompatibility leads to a conflict between the representation of the action’s resident effect (the body movement) and its remote effect (the object movement). In order to carry out an action, representations of the effects of an action need to be activated, which then in turn activate the appropriate motor commands. Since both, representations of body movements and of visual effects of these movements, can be used to perform actions (Pfister, 2019), humans could strategically suppress some conflicting representations to reduce conflict (e.g., Heuer & Rapp, 2012; Wirth, Pfister, Brandes, & Kunde, 2016). This mechanism could thus be another possible explanation for the absence of action–effect binding in the inverted condition. In particular, the strong conflict between hand and cursor movement during movement might have prompted the participants to suppress the irrelevant cursor position when judging the stylus position. Support for this assumption also stems from our findings that reaction times were significantly slower in the inverted (incompatible) condition than in the additive (compatible) condition, which reflects this conflict during movement planning. Whether and to what extent this conflict is still present at later stages of the movement and during the spatial judgments is something we aim to address in future research. Nevertheless, we believe that the ability of our paradigm to show such conflict and how it possibly relates to the elimination of spatial binding through suppression of conflicting sensory signals is a valuable extension to the field.

While we focused our analyses on linear relationships, the figures and exploratory analyses suggested a contribution of nonlinear components. Linear relations are typically assumed as a kind of default in studies on spatial binding and multisensory integration (Debats & Heuer, 2018a, 2018b, 2018c; Kirsch et al., 2016; Rand & Heuer, 2013; Rand & Rentsch, 2016; Rand et al., 2013). Nonlinear relations suggest a kind of breakdown of integration with sufficiently large distances of hand and cursor. While the linear component captures the main variance and thus our underlying research question (with nonlinear aspects not consistently improving the fit significantly), it would certainly be interesting to study in more detail the shape of the drift curve under various conditions.

In past research, proprioceptive drift has generally been interpreted as a marker for an (implicit) sense of ownership over remote objects, thus the feeling how much a body-external object tends to be integrated into one’s own body (Botvinick & Cohen, 1998; Kalckert & Ehrsson, 2012). While originally observed and studied in the rubber-hand illusion, proprioceptive drift has by now been reported to emerge with other noncorporeal objects as well (e.g., Armel & Ramachandran, 2003; Ma & Hommel, 2015). One could argue that the actual incorporation of an arbitrary object into the body seems unlikely and should not be compared with the proprioceptive drift observed with a rubber hand or virtual hand, which seems more “realistic” to be actually experienced as belonging to one’s own body because it actually resembles the anatomy of the human hand (Guterstam, Gentile, & Ehrsson, 2013; Kalckert, Bico, & Fong, 2019). However, given the increasing number of studies showing proprioceptive drift also for anatomically nonplausible objects, it seems tenable to assume that artificial, noncorporeal objects can at least lead to a shift of the body representation towards these objects since this is essentially what proprioceptive drift measures.

Whether or to what extent the subjective experience of agency and ownership for the cursor were also affected similarly by the different kinds of movement transformations is questionable, though. In fact, we found little evidence that ownership ratings were affected by the different kinds of movement transformations at all. It has to be noted, though, that overall explicit ownership ratings were very low, so it is questionable if or to what extent these reflect actual, conscious experiences of ownership of the cursor, regardless of possible differences or lack of differences between conditions (see also Kalckert, Bico, & Fong, 2019, for similar arguments).

However, and especially against this background, the inconsistencies between the implicit binding measure and the explicit ratings are an interesting finding that add up to an increasing number of studies showing that implicit measures of agency and ownership, such as temporal or spatial binding, might be related to explicit sense of agency and ownership to a lesser extent than previously thought (Buehner, 2012; Kirsch et al., 2019; Majchrowicz & Wierzchoń, 2018; Moore et al., 2009; Schwarz et al., 2019; Suzuki et al., 2019). While we cannot rule out possible methodological reasons for our inconsistent findings, disentangling the relationship between explicit agency and ownership experiences and different binding measures further could be an interesting question for future research.

## Conclusions

Spatial action–object binding, an indication of implicit object ownership, can be obtained with actively controlled noncorporeal objects that show little, if any, similarity to the human body. However, immediate control over an object is not sufficient to induce implicit ownership. If the object moves into a direction that is in conflict to the movement of the controlling effector, then no spatial binding occurs, possibly due to suppression of conflicting visual representations.
